# Giant Slalom: Analysis of Course Setting, Steepness and Performance of Different Age Groups — A Pilot Study

**DOI:** 10.3389/fspor.2020.00107

**Published:** 2020-08-26

**Authors:** Björn Bruhin, Rowie J. F. Janssen, Sebastien Guillaume, Mara Gander, Felix Oberle, Silvio Lorenzetti, Michael Romann

**Affiliations:** ^1^Swiss Federal Institute of Sport Magglingen (SFISM), Magglingen, Switzerland; ^2^Institut d'ingénierie du territoire, Haute Ecole d'Ingénerie et de Gestion du Canton de Vaud, Yverdon-les-Bains, Switzerland; ^3^Sports Medical Research Group, Department of Orthopaedics, Balgrist University Hospital, University of Zurich, Zurich, Switzerland

**Keywords:** giant slalom, youth sports, course setting, performance, elite sports

## Abstract

**Introduction:** Giant slalom is the core discipline of alpine skiing, and each race has its own specific course and terrain characteristics. These variations may explain differences in the speed and time per turn profiles, which are essential for performance development and injury prevention. This study aims to address the differences in course setting and steepness of the different course sections (flat—medium—steep) and compare them to the performance parameters among young (U12, U14, U16) and older (U18, U21, elite) male athletes.

**Methods:** The study examined a total sample size of 57 male athletes; 7 from elite level, 11 from U21, 13 from U18, 6 from U16, 13 from U14, and 7 from U12. The athletes wore a portable global navigation satellite system (GNSS) sensor to extract performance parameters. The course profiles and gate positions of nine runs were measured with differential GNSS. The runs were divided into flat, medium and steep sections. From the performance parameters (speed, time per turn, etc.) and the course setting variables, the mean value per section was calculated and used for the further analysis.

**Results:** In total, 192 run sections from 88 runs were recorded and analyzed. Comparisons between course settings in young and older classes showed no significant differences. However, the turning angles and horizontal gate distances were smaller in flat sections. Average speed (49.77 vs. 65.33 km/h) and time per turn (1.74 vs. 1.41 s) differed significantly between young and U21/elite categories. In medium terrain sections U21 and elite athletes spent more time in the gliding phase compared to all other athletes.

**Discussion:** It seems to be a reasonable that, given similar course setting and steepness, speed increases concurrently with the technical and tactical skills of the athlete. Moreover, the finding that the elite athletes spent more time in the gliding phase could be crucial for understanding technique and performance development in young athletes.

## Introduction

The four main disciplines of the World Cup of Alpine Ski Racing are slalom, giant slalom (GS), super-G and downhill. Because GS is the core discipline of alpine skiing, it was the exclusive focus of this study. Each race has its own specific course and terrain characteristics regulated by the International Ski Federation (FIS). Nevertheless, the course setting can vary greatly since the FIS only defines certain boundaries, such as the number of gates and vertical drop (VD). For example, the horizontal distance between two successive gates is not defined and appears to increase with terrain inclination (Gilgien et al., 2015). In addition, there is a slight trend toward a shorter direct gate-to-gate distance for the section of the race that increases in inclination; thus, in the flat sections, the courses are set straighter than in the steep sections for which a more sinuous course setting is usually chosen (Bruhin et al., 2018). These characteristics influence the speed of the athlete and are also associated with a decrease in speed as steepness increases as well as increased horizontal distances and shorter direct gate-to-gate distances. Spörri et al. (2012a) revealed that these characteristics can produce greater time differences among athletes than other characteristics as e.g., vertical gate distance. Moreover, course setting and terrain inclination contribute to 57% of the skier speed (Gilgien et al., 2015).

It is essential to understand course setting because it can be related to the incidence and severity of injuries in athletes. In an attempt to reduce speed, an increase in horizontal gate distance has been proposed. As Spörri et al. (2012a) revealed, this may decrease speed but it is not the best way to reduce injury incidence. Furthermore, an increased horizontal gate distance can induce fatigue as a consequence of loading forces acting for a longer duration, which tends to be the greatest upon the completion of a turn. In addition, a greater horizontal gate distance may increase the risk of off-balance situations by forcing the athlete to fatigue both the backward and inward leaning spectrum. Thus, it might be of greater benefit to an athlete's safety to locally slow down a racer for hazardous points of the course (e.g., terrain changes, key sectors). Additionally, the vertical gate distance should also be considered as this causes a decrease in skier's speed without the aforementioned drawbacks (Gilgien et al., 2020).

The FIS sets rules for course settings in GS. 11 to 15% of the VD (250 to 450 m) encompasses the number of directional changes for elite races (International Competition Rules (ICR) and Fédération Internationale de Ski (FIS), 2019). A greater number of regulations are specified for young athletes and are homogenous for the U16, U14, and U12 groups. Moreover, GS gates must be placed as follows: the distance between open gates is 22 ± 5 m with a maximum of three gates, including delayed gates, at a maximal distance of 35 m. At the delayed gates, a minimum distance of 15 m between the two consecutive gates is required. Additionally, the VD for young athletes should not exceed 300 m (National Competition Rules (NCR) and Swiss-Ski, 2019).

The National Federation of Switzerland has established specific rules regarding the course setting for young athletes. Before entering the elite competition, skiers are divided into the following age groups: U12, U14, U16, U18, and U21. It should be noted that there are no differences in course setting applied to these groups (National Competition Rules (NCR) and Swiss-Ski, 2019). The young athletes included in this study received coaching, feedback and an intense level of training and competition (Romann and Fuchslocher, 2014). Overall, while all of these factors enhance performance, it is known that intensive ski training is physically demanding, and there is a high risk of injury independent of age and gender (Spörri et al., 2017). To prevent this, a specific training regimen should be incorporated (Müller et al., 2017), and knowledge of performance and course setting becomes more valuable.

In addition to injury prevention, a progressive transition for young athletes to the elite level is important; therefore, specific data on each age category are necessary for coaches and race organizers. Since course setting has a major influence on the athlete's speed, it is essential to thoroughly examine these characteristics (Spörri et al., 2012a; Gilgien et al., 2020). The FIS sets regulations on the slope and course settings for elite, U21 and U18 athletes, and the National Federation specifies young race conditions.

To our knowledge there is no study which compares the course conditions (course setting, steepness) according to the aforementioned rules in young vs older athletes. Neither a study which focuses on the differences on the performance (e.g., turn phases, speed, time per turn) of young vs. older athletes. Therefore, the aim of the present study was to identify differences in sections of the runs (course setting, steepness) and performance parameters (e.g., speed, time per turn, turn phases etc.) among young and older athletes. The application of these procedures is predicted to improve knowledge about performance development and injury prevention in the context of GS.

## Materials and Methods

### Participant Characteristics

Measurements were taken for six age levels, which included a total sample size of 57 male athletes; seven were from the elite level (all of the elite athletes were older than 21 y), 11 were from the U21 level, 13 were from U18, 6 were from U16, 13 were from U14, and 7 were from U12. The elite skiers were born in 1992 ± 2.18 y and had a mean world ranking of 49 ± 69.02 (min: 5; max: 152). In accordance with the rules of the international federation (International Competition Rules (ICR) and Fédération Internationale de Ski (FIS), 2019), the groups elite, U21 and U18 are combined in some evaluations to “older group” and the groups U16, U14, and U12 as JO (Abbreviation from Swiss Ski Federation: Jugend Organization = youth organization) group. Especially for the race analysis this makes sense, because the boys participated together in the JO races and the older group in the FIS races.

### Race Description

The measurements were taken at three different locations during the 2018–2019 and 2019–2020 winter seasons. In Hoch-Ybrig (Schwyz, Switzerland) at the Swiss National Championships on March 23–24, 2019 and a FIS race on January 14, 2020. The VD for this hill is officially recorded as 295 m. In Les Diablerets (Vaude, Switzerland) at a FIS race on February 8, 2020 with a VD of 340 m. In Meiringen (Bern, Switzerland) at regional races from January 19, 2019 and January 26, 2020 and the VD for this hill is 264 m. With the exception of the race in Meiringen on January 26, 2020, all races consisted of two runs. Table 1 shows the included athletes per age group and per race.

**Table 1:** Athletes and sections of all competitions.

		**2019_HY_run1**	**2019_HY_run2**	**2020_HY_run1**	**2020_HY_run2**	**2020_LesD_run1**	**2020_LesD_run2**	**2019_M_run1**	**2020_M_run1**	**2020_M_run2**	**Total**
Number of athletes per run	Elite	4	4	2	1	1					12
	U21			5	5	6	4				20
	U18			5	7	5	3				20
	U16							3	1	3	7
	U14							8	4	5	17
	U12							2	5	5	12
	Total	4	4	12	13	12	7	13	10	13	88
Number of sections per run		3	3	3	3	3	3	1	1	1	21
Turns per section	Flat	17	17	21	21	10	10				96
	medium	19	19	17	17	16	16	42	37	36	219
	steep	6	6	4	4	19	19				58
Total number of mean values per race		12	12	36	39	36	21	13	10	13	192

*HY, Hoch-Ybrig; LesD, Les Diablerets; M, Meiringen*.

### Course Setting Parameters

Each gate was measured with a global navigation satellite system (GNSS) device in differential mode (Gilgien et al., 2015). The geodetic coordinates of the gates were determined in post-processing with L1 carrier-phase data, carried out with two u-blox M8 GNSS receivers. One receiver was used as static reference. The second was used as rover (baseline <2 km). The differential fixed ambiguities carrier-phase post-processing was carried out with the Free and Open-Source Software (FOSS) RTKLIB v.2.4.2 in order to achieve an accuracy of 5 cm.

All course setting parameters were calculated based on the coordinates of each gate (Gilgien et al., 2015). The gate distance is the linear distance between two gates. The horizontal gate distance is defined as a perpendicular line from one gate to the lines between the previous and subsequent gates. The vertical gate distance describes the distance from one gate to the beginning of the perpendicular line. The turning angle is calculated as the angle between the extended lines from two consecutive gates to the line of the gate to the next gate, according Gilgien et al. (2015). The steepness is defined as the difference between two consecutive turns and the section steepness is the average value per section.

### Performance Parameters

**Figure 1:**
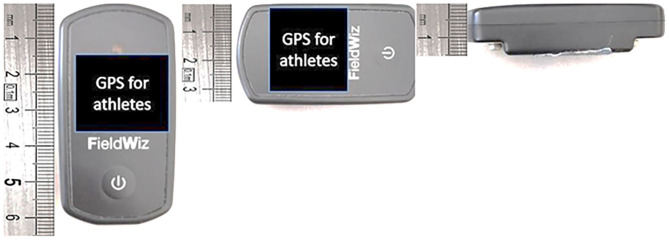
Details of the GNSS Sensor; dimensions: 65 × 35 × 15 mm; weight: 35 g.

All athletes had a portable GNSS sensor on their back protectors. The sensor is shown in Figure 1 and was attached to the back protector with double-sided adhesive tape at the height of the third thoracic vertebrae. For safety reasons, it was placed next to the spine. To address to the performance parameters, the speed (km/h) information and trajectory of the entire run were analyzed.

#### Calculated Variables

**Figure 2:**
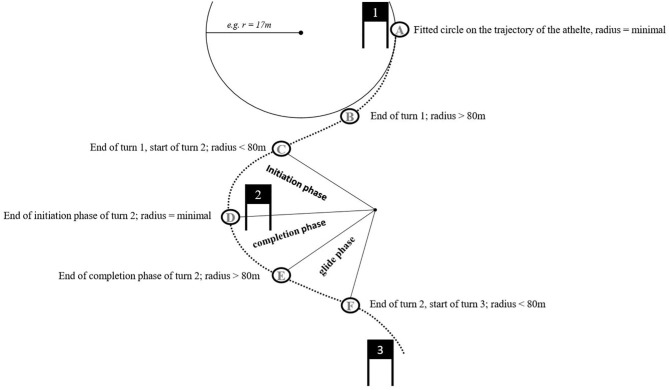
Definition of characteristic points in a skier's trajectory and turn phases.

In collaboration with expert coaches, a turn was defined as the start of a turn to the start of the next turn. The start of a new turn was set when the fitted radius first dropped below 80 m. The minimal turn radius was set as the apex of the turn, and the end of the turn was determined as the point at which the turn radius exceeded 80 m again for the first time (Figure 2). The start of the turn to the minimal turn radius was identified as the initiation, the minimal turn radius to the end of the turn was identified as the completion and the end of the turn to the start of the next turn was identified as the gliding phase. The relative amounts of time spent in the respective turn phases from each athlete during the entire turn were calculated. These ratios were averaged along the entire run.

Based on the fitted trajectory of the athlete, several parameters of one turn [time of the start of the turn (s), minimal turn radius (m) and time of the end of the turn (s)] were calculated. Based on these defined points, the per-turn calculations could be conducted for the following variables: (a) the decrease in speed during the initiation phase and (b) the speed increase from the minimal turn radius to the beginning of the next turn. Both were expressed as a percentage of the start speed in the specific turn.

### Parameter Computation

In total, 88 runs were measured, and each run was analyzed as an individual measurement. Twenty-six athletes performed one run, 31 athletes performed two runs. At each of the nine races, the track profile was analyzed. According to conspicuous terrain transitions the sections of the run were divided into three different categories (flat, medium, steep) (Bruhin et al., 2018). The section was assigned to “flat” if the average steepness of the section was lower than 13.5°, to “medium” if the average steepness of the section was between 13.5° and 18.9° and to “steep” if the average steepness of the section was above 18.9°. The results of this division can be seen in Table 2. A total of 21 sections were analyzed. All the athletes' turns within one section of a course were averaged. These average values per section and per athlete were treated as measured values in the subsequent analysis.

**Table 2:** Course setting parameters (average and standard deviation) per run and terrain section.

	**Flat**	**Medium**	**Steep**	**Older group_medium**	**JO_medium**	**2019_M_JO_ boys**	**2020_M_JO_run1**	**2020_M_JO_run2**	**2020_LesD_FIS_run1**	**Flat**	**Medium**	**Steep**	**2020_LesD_FIS_run2**	**Flat**	**Medium**	**Steep**
Gate distance (m)	27.08	25.34	26.08	26.32	25.07	26.87	23.67	24.29	26.35	25.01	25.49	27.57	25.40	25.75	24.98	25.56
Std	1.45	1.78	2.37	3.68	3.67	2.34	3.01	4.61	3.14	2.76	4.14	1.46	4.54	5.84	4.00	4.17
Steepness (°)	−11.96	−16.24	−20.62	−15.29	−16.60	−14.66	−17.84	−17.72	−16.91	−12.95	−14.60	−20.53	−16.85	−12.95	−14.66	−20.55
Std	1.40	1.09	1.04	6.45	5.19	4.97	5.10	4.81	5.81	7.25	5.30	2.31	5.51	6.10	4.86	2.68
Turning angle (θ)	25.58*/**	31.51*	33.48**	31.87	32.23	31.70	32.42	32.69	29.67	26.51	28.43	32.03	29.50	25.26	27.97	32.54
Std	1.93	1.74	1.19	10.87	9.35	8.36	9.41	10.36	8.21	11.55	7.39	6.01	8.24	10.45	7.22	6.49
Vertical gate distance (m)	25.88	23.96	24.94	25.05	23.98	25.78	22.61	23.15	25.30	23.06	25.07	26.47	24.65	25.09	24.56	24.49
Std	1.47	1.11	2.30	3.86	3.66	2.43	3.02	4.54	3.37	3.98	3.97	1.56	4.50	5.80	3.93	4.14
Horizontal gate distance (m)	5.81*/**	6.89*	7.36**	7.18	6.92	7.28	6.61	6.80	6.70	5.62	6.20	7.55	6.26	5.08	5.96	7.08
Std	0.51	0.35	0.55	2.67	2.21	1.89	2.05	2.64	2.11	2.77	1.98	1.40	2.25	2.86	1.79	1.86
	**2019_HY_FIS_run1**	**flat**	**medium**	**steep**	**2019_HY_FIS_run2**	**flat**	**medium**	**steep**	**2020_HY_FIS_run1**	**flat**	**medium**	**steep**	**2020_HY_FIS_run2**	**Flat**	**Medium**	**Steep**
Gate distance (m)	26.15	27.27	25.08	25.83	26.55	27.19	26.97	25.55	27.58	28.30	26.60	28.32	27.85	28.99	27.50	23.64
Std	1.02	1.35	0.98	0.72	1.18	3.35	0.98	0.75	2.98	1.80	3.87	1.63	3.54	2.58	2.01	7.31
Steepness (°)	−13.81	−11.39	−14.29	−19.07	−14.66	−11.50	−15.67	−20.41	−14.83	−11.31	−17.35	−21.72	−14.73	−11.66	−16.58	−21.45
Std	6.39	3.89	7.43	4.61	4.97	3.80	3.91	4.32	7.13	7.02	5.20	4.82	6.80	4.82	7.27	4.85
Turning angle (θ)	26.66	22.27	28.81	33.76	31.70	28.55	33.47	35.85	30.06	24.70	36.46	32.59	30.71	26.21	35.48	34.09
Std	11.07	7.68	13.01	7.66	8.36	6.87	9.07	6.72	10.19	8.95	8.62	4.98	11.14	12.12	7.52	8.73
Vertical gate distance (m)	25.29	26.69	24.01	24.72	25.78	26.33	25.73	24.27	25.80	26.06	25.12	27.17	26.85	28.06	26.42	22.52
Std	3.38	1.37	4.59	1.24	2.43	3.25	1.37	0.78	4.50	5.03	4.15	1.34	3.55	2.67	1.65	7.25
Horizontal gate distance (m)	6.08	5.25	6.55	7.17	7.28	6.64	7.75	7.84	6.90	5.68	8.24	7.91	7.31	6.58	8.39	6.59
Std	2.49	1.86	2.90	2.04	1.89	1.65	2.04	1.47	2.49	2.32	2.06	1.50	2.76	3.01	1.86	3.00

In the first three columns the average and standard deviation per category (flat, medium, steep) for all races are shown. Significant (p < 0.05) differences between groups are marked with an asterisk (*flat vs medium, **flat vs steep). HY, Hoch-Ybrig; LesD, Les Diablerets; M, Meiringen.

### Statistics

The Levene's test assessed the equality of variances. Afterwards, for all results, a one-way analysis of variance (ANOVA) was performed to identify any significant differences. Finally, as *post-hoc* tests, the Student's *t*-test with Bonferroni correction was used for equal and unequal variances while for unequal variances, the Welch's *t*-test was selected. All tests were conducted in SPSS (IBM SPSS Statistics for Windows, Version 25.0, Armonk, NY, 2017).

## Results

### Course Setting Parameters

In total, 192 run sections from 88 runs were recorded and analyzed. Comparisons in course setting between JO and the older classes were only made in the medium sections, due to the different number of observations. No significant difference was found between the JO and the older group.

The average values of the different section categories for all competitions are shown in Table 2. The sections differed in steepness, and the turning angle was smaller in the flat condition compared to the medium and steep condition. In addition, the horizontal gate distance in flat conditions was smaller than in medium and steep conditions where gates are set with a larger horizontal offset. No significant difference in the course setting parameters could be detected between the steep and medium conditions.

Regarding U18, U21 and elite competitions in steep terrain (Table 3); the gate-gate distance as well as the vertical gate distance were significantly shorter in the elite races (gate-gate distance = 25.7 m; vertical gate distance = 24.9 m) than in the races of the U21 and U18 group (gate-gate distance = 27.3 m; vertical gate distance = 26.2 m). Therefore, the turning angle did not differ significantly.

**Table 3:** Course setting parameters (average and standard deviation) per age group and terrain section.

	**Elite**	**FIS**	**JO**	**Elite (flat)**	**Elite (medium)**	**Elite (steep)**	**FIS (flat)**	**FIS (medium)**	**FIS (steep)**	**JO (medium)**
Gate distance (m)	26.35	27.02	25.07	26.87	26.08	**25.69**	27.23	26.49	**27.31**	25.07
Std	2.48	3.49	3.67	1.37	3.42	0.75	3.60	3.84	2.78	3.67
Steepness (°)	−14.23	−15.88	−16.60	−11.45	−14.96	−19.74	−12.89	−15.53	−20.70	−16.60
Std	5.75	6.41	5.19	3.84	6.02	4.52	5.93	6.74	2.91	5.19
Turning angle (θ)	29.21	29.94	32.23	25.41	31.21	34.81	26.70	32.34	32.25	32.23
Std	10.11	9.44	9.35	7.93	11.40	7.28	9.52	10.45	6.23	9.35
Vertical gate distance (m)	25.54	25.89	23.98	26.51	24.89	**24.49**	26.20	25.16	**26.20**	23.98
Std	2.94	3.86	3.66	2.50	3.46	1.06	4.20	4.11	2.80	3.66
Horizontal gate distance (m)	6.68	6.85	6.92	5.95	7.15	7.50	6.15	7.20	7.50	6.92
Std	2.29	2.41	2.21	1.89	2.58	1.81	2.43	2.73	1.63	2.21

*Significant (p < 0.05) differences between groups are marked in bold (= elite vs. FIS, JO)*.

### Performance Parameters

The JO group only had medium terrain sections and in order to obtain comparability among the younger and older athletes, they are compared in the medium terrain section. The older athletes are compared in all three sections.

#### All Athletes in Medium Terrain Section

The time needed per turn (Table 4) differed between the defined age groups in a range of 1.74 s (U12) and 1.41 s (elite). The elite group had the lowest time per turn and differed significantly from the three young groups (U12, U14, and U18). The youngest group (U12) needed significantly the most time per turn, compared to all groups. Furthermore, the U14 group had a significantly higher time per turn than the U21 group.

**Table 4:** Performance parameters (average and standard deviation); time per turn (s) and speed (km/h) for all groups in the medium terrain sections.

		**U12**	**U14**	**U16**	**U18**	**U21**	**Elite**
Time per turn (s)	Mean	1.74^#+!!″‡^	1.58^*″‡^	1.56*	1.55^*‡^	1.49^*#^	1.41^*#!!^
	Std	0.10	0.11	0.08	0.09	0.07	0.13
Speed (km/h)	Mean	49.77^#+!!″‡^	54.00^*+!!″‡^	55.60^*#!!″‡^	62.13^*#+″^	65.33^*#+!!^	61.30^*#+^
	Std	1.91	1.23	1.13	2.71	3.02	4.77

Significant (p < 0.05) differences between groups are marked by group-specific markers (U12^*^; U14^#^; U16^+^; U18^!!^; U21″; Elite^‡^).

The average speed range (Table 4) was between 49.77 km/h (U12) and 65.33 km/h (U21). The older group showed a significantly higher average speed than the JO group. In addition, the U21 group had a significantly higher speed than the U18 group. Also, the U16 exhibited a significantly higher average speed than the U14 and the U12 age groups and the U14 reached a higher speed than the U12 group.

**Figure 3:**
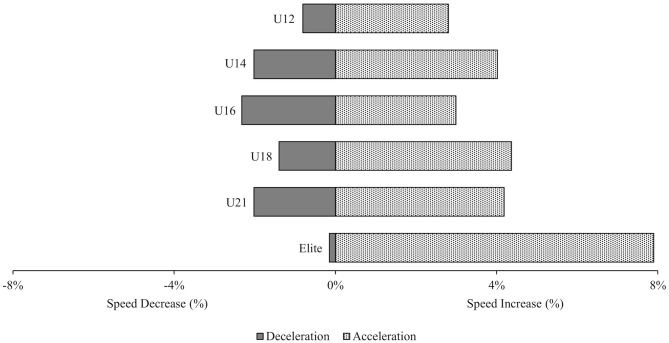
Mean percentage of the difference in speed between two turn phases in the medium terrain section; the acceleration phase refers to the section from the minimal radius to the start of the next turn; the deceleration phase is from the start of the turn to the minimal radius.

Speed gain and speed decrease between two turn phases are displayed in Figure 3. No significant differences were found in the acceleration and deceleration phase, respectively.

**Figure 4:**
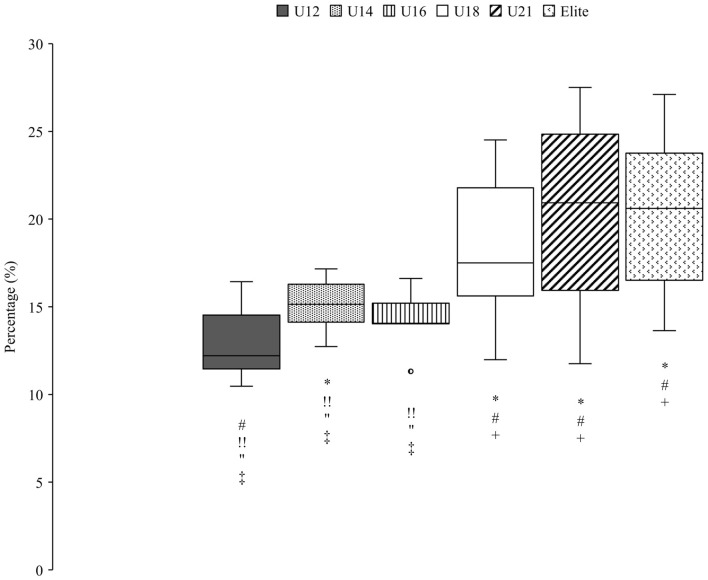
Percentage of all groups in the gliding phase of the medium terrain section, expressed as a percentage of the total run time. Significant (*p* < 0.05) differences between groups are marked by group-specific markers (U12*; U14^#^; U16^+^; U18^!!^; U21″; Elite). Outliers are shown with a circle, filled with the color of the corresponding group.

Relative time spent in the different turn phases of all groups are shown in Table 5, Figure 4. The youngest group (U12) spent significantly more relative time in the initiation phase than the U14 and the elite group. Older athletes spend less time in the completion phase and more in the gliding phase compared to the JO group in the medium terrain section. With the exception between U18 and U16, all results were significant in the completion phase. Furthermore, the gliding phase differed significantly between the JO and the U18, U21, elite groups.

**Table 5:** Mean percentage for all groups in medium terrain for the initiation, completion and gliding phases, expressed as a percentage of the total turn cycle.

		**U12**	**U14**	**U16**	**U18**	**U21**	**Elite**
Initiation phase (%)	Mean	44.02^#″^	41.22*	43.70	42.67	40.93*	41.85
	Std	2.27	2.61	4.05	3.02	3.18	3.21
Completion phase (%)	Mean	43.10^!!″‡^	43.58^!!″‡^	42.02^″‡^	39.20^*#^	38.44^*#+^	37.83^*#+^
	Std	1.54	2.10	3.05	2.10	2.74	2.16
Gliding phase (%)	Mean	12.88^#!!″‡^	15.19^*!!″‡^	14.28^!!″‡^	18.12^*#+^	20.62^*#+^	20.32^*#+^
	Std	1.84	1.36	1.60	3.76	4.90	4.22

Significant (p < 0.05) differences between groups are marked by group-specific markers (U12^*^; U14^#^; U16^+^; U18^!!^; U21″; Elite^‡^).

#### Older Athletes in Three Terrain Sections

**Figure 5:**
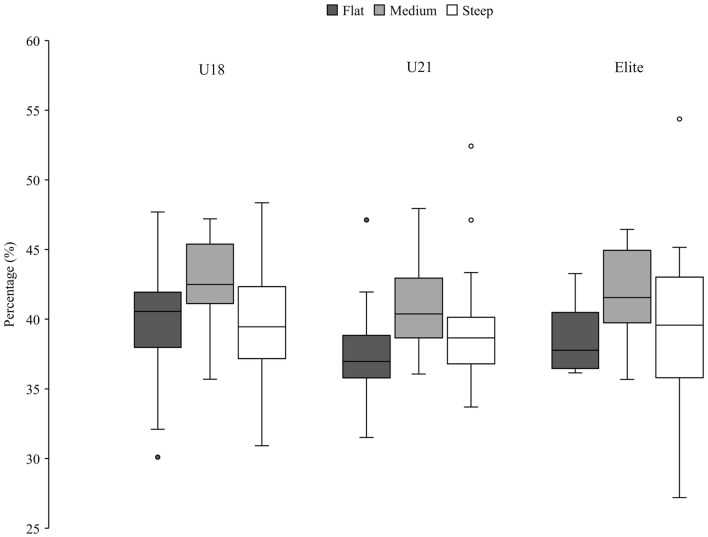
Percentage of the U18, U21 and elite group in initiation phase, expressed as a percentage of the total run time. No (*p* < 0.05) significant differences were found among these three groups. Outliers are shown with a circle, filled with the color of the corresponding group.

**Figure 6:**
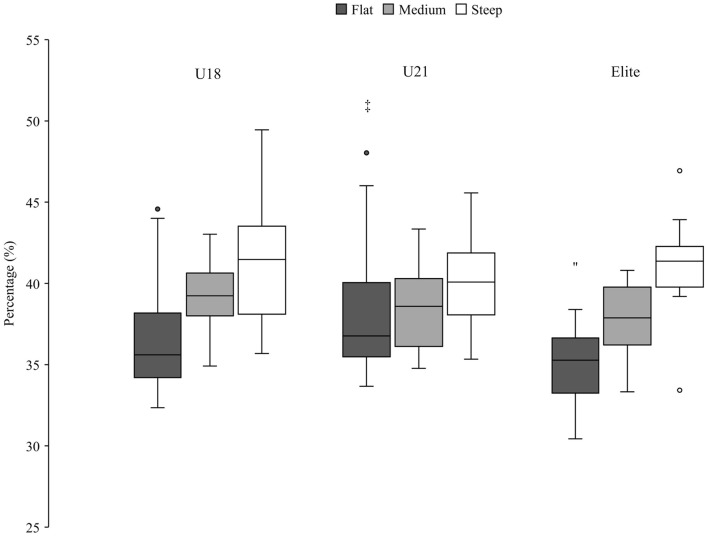
Percentage of the U18, U21 and elite group in the completion phase, expressed as a percentage of the total run time. Significant (*p* < 0.05) differences between groups are marked by group-specific markers (U18^!!^; U21″; Elite). Outliers are shown with a circle, filled with the color of the corresponding group.

**Figure 7:**
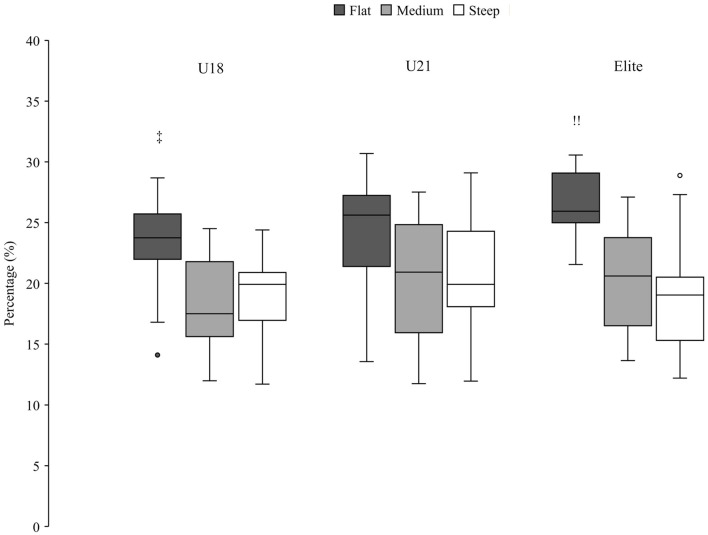
Percentage of the U18, U21 and elite group in the gliding phase, expressed as a percentage of the total run time. Significant (*p* < 0.05) differences between groups are marked by group-specific markers (U18^!!^; U21″; Elite). Outliers are shown with a circle, filled with the color of the corresponding group.

Zoomed in on the older group, no significant differences were found in the initiation phase in the three terrain sections (Figure 5). In the completion and gliding phase, significant differences were found in the flat terrain section. Elite athletes spent less relative time in the completion phase than the U21 group (Figure 6) and more time in the gliding phase compared to the athletes from the U18 group (Figure 7).

## Discussion

The main finding of the study was that average course settings in young classes (U12, U14, U16) did not differ in comparison with the older classes (U18, U21, elite). However, compared to the medium and steep sections, the turning angles and horizontal gate distances were shorter in the flat sections. Average speed for the medium terrain section was between 49.77 km/h (U12) and 65.33 km/h (U21) and time per turn differed significantly between 1.74 s (U12) and 1.41 s (elite). Additionally, differences were found in the mean speed and turn phase parameters, more precisely in the gliding and completion phases. In medium terrain sections U21 and elite athletes spent more time in the gliding phase compared to all other athletes.

A “high skiing speed” is commonly called a major course-related injury factor (Spörri et al., 2017). As stated in the literature (Spörri et al., 2012a), shorter linear gate distances and higher steepness correlate with decreased speed. Furthermore, it is supported by previous research that an increased horizontal distance is associated with decreased speed (Spörri et al., 2012a). Spörri et al. (2017) have also postulated that an increased horizontal gate distance is associated with a potentially increased risk of injury. This is provoked by increased fatigue and greater risk of “out-of-balance” situations.

When compared to the older athletes, the JO group exhibited significantly lower velocities and longer time per turn, but the horizontal gate distance was not significantly larger. Gilgien et al. (2015) stated that 57% of a skiers speed can be explained by course setting and terrain inclination. The technical skills of the older athletes were significantly better compared to the JO group. In addition, the older group was longer in the gliding phase. It seems to be a reasonable relation that with similar course setting and steepness, the speed increases with the technical and tactical skills of the athletes.

In this context the course setting in the measured races can be critically undermined. It seems that the extremes of development (U12 and elite) should be more clearly differentiated in order to achieve a skill transfer and to reduce injury risk. Furthermore, it can be discussed whether interventions should be taken to adjust the course setting in both levels. In the elite's course setting our results support Spörri et al. (2012a), that a larger horizontal distance is proposed to reduce the speed. In the JO's course a shorter gate distance is proposed to reduce the time per turn.

### Course Setting Parameters

Gilgien et al. (2015) measured the course setting and terrain characteristics during seven male World Cup races in the winter seasons of 2010–2011 and 2011–2012, with a total of 14 runs. Gate-gate distance was recorded as 26.24 ± 2.25 m, horizontal gate distance was 7.47 ± 2.93 m and steepness was 17.80° ± 7.0° (Gilgien et al., 2015). Compared to the present findings of the older groups, it can be concluded that the horizontal distance of 6.79 ± 2.38 m, steepness of −15.34° ± 6.25° and gate-gate distance of 26.80 ± 3.21 m were comparable to the values in the literature. However, the course setting values for the JO athletes were slightly different (gate-gate distance 25.07 ± 3.67 m; horizontal gate distance 6.92 ± 2.21 m) than the values of the older group (gate-gate distance 26.32 ± 3.68 m; horizontal gate distance 7.18 ± 2.67 m) in the medium terrain section. This follows the literature since stricter regulations are applied for these age groups (National Competition Rules (NCR) and Swiss-Ski, 2019).

The horizontal gate distance for the older groups in flat conditions (5.81 ± 0.51 m) was smaller than in medium (6.89 ± 0.35 m) and steep conditions (7.36 ± 0.55 m), where gates were set with a larger horizontal distance. The turning angle is described as the gate-gate distance, the vertical gate distance and the horizontal gate distance. Since there were neither significant differences in the gate distance, nor in the vertical gate distance, it can be stated that the turning angle is mainly influenced by the horizontal distance.

### Performance Parameters

Besides differences in the course profile, the time the athletes needed per turn in the medium terrain section decreased with higher age groups (Table 4). The U12 group needed 1.74 ± 0.1 s and the elite athletes 1.41 ± 0.13 s. When comparing the results of the elite group to an earlier work of Spörri et al. (2012b); an average of 1.41 s/turn in the present study for the elite athletes compared to 1.68 s/turn (Spörri et al., 2012b), a trend to a smaller time per turn can be observed. The decrease in time per turn can be explained by the constant course setting. Although the turning angles and the horizontal gate distance were larger, these parameters did not differ significantly between the JO and older group. However, speed differed significantly in the medium terrain section and therefore it can be assumed that a similar distance is traveled at a higher speed and therefore the time per turn must be smaller. The decreasing trend in time per turn and increasing trend of higher speed indicate evidence of smooth skill transfers from the young athlete level to the elite level.

The difference in speed gain and speed decrease in the medium terrain section was not significant between any group but there seemed to be a tendency in elite athletes to brake less hard to the center of the turn and then take more speed out of the turn than all younger athletes (Figure 3). However, as mentioned, these differences are not significant and needs to be tested with a larger sample.

It is notable that the elite athletes did not have the highest speed of all groups in the medium terrain section. The lowest average speed was reached by the U12 athletes, the highest speed by the U21 athletes and in between there was an increasing trend (Table 4). Importantly, it should be mentioned that the sections were in the same category (medium) but did not have the same inclination. The sections of the competitions (Table 3) in which the elite athletes participated (−14.29°; −15.67°) were below the average value of all races (−16.24° ± 1.09°).

The changes in speed during turns in the medium terrain section was not significant between the groups (Figure 3). Nevertheless, a slight trend could be observed, the elite group had a small speed decrease to the minimal radius and a high increase of speed to the next turn. Supej et al. (2011) suggested a “speed barrier” as a possible explanation for this. Elite athletes try to increase their speed after the apex of the turn and braked less before the apex of the turn to reach the goal of maintaining the highest speed possible. With a larger horizontal offset, the speed decreases prior to the apex of the turn should be larger (Spörri et al., 2012a). In one study (Spörri et al., 2012a), this larger offset was seen to potentially increase fatigue in the athlete. In terms of the present study's results, it can be assumed that higher speed variations are more demanding for the athletes and will increase their fatigue.

The gliding phase (Table 5) was longest in the older groups than in the JO group. Thus, a closer look was taken at the two turn phases initiation and completion. It was shown that the older athletes compensated the longer gliding phase with a shorter completion phase. Few differences were found in the initiation phase.

This study is the first to calculate tactical parameters, such as relative time in each turn phase, in this way. Spörri et al. defined the segments of the turn differently (Spörri et al., 2012a); due to a lack of information about the skier's trajectory, the turn segments could not be defined in the same way and required a new definition. It should be noted that in an earlier study (Spörri et al., 2012b) a similar initial phase (and then a shorter final phase) led to better performance as well. Moreover, in the present study there was a difference between the older group and the JO athletes in terms of gliding time. Compared to the JO group, the older groups, spent less time in the completion and more time in the gliding phase. In Figures 6, 7 the older groups are shown in the different terrain sections. It is notable that the same pattern appeared in flat, medium and steep sections. The gliding phase was longest in the flat terrain, shortest in the steep terrain and in between in medium terrain (Figure 7). The reverse pattern could be seen in the completion phase (Figure 6). The longest completion phase was in steep terrain, the shortest completion phase was in flat terrain and in between values for completion phase in the medium terrain sections.

### Limitations

All terrain sections in the course of the JO group were labeled as medium which restricted us to make comparisons between JO and the older groups in the flat and steep terrain sections. Overall, the findings of this pilot study should be confirmed by observing identical athletes in a repeated measurement design. This would lead to a greater number of measurements and allow a turn by turn analysis to confirm these outcomes.

Course setting and performance parameters were measured with a GNSS device. The use of a geodetic, multi-frequency receiver providing differential position solutions could result in greater accuracy. Due to the limited amount of time between the runs and the time-consuming aspect of acquiring data for the course setting parameters, this was not applicable in the present study. Gløersen et al. (2018) compared these systems and concluded that the present methods were inferior, but typical, instantaneous speed differences could be obtained with a maximal horizontal error of 2.09 m, a maximal vertical error of 2.71 m and a time precision of 0.30 s. However, an inverse relationship was found between measurement error and skiing speed which indicates larger errors at lower speeds. No low speeds were observed during the runs and the given error was overestimated in the present study.

## Conclusion

The current study showed no differences in course setting between young and older groups. However, differences were found in the mean speed and turn phase parameters, more precisely in the completion and gliding phases. Results of this analysis form a basis to critically discuss the course setting, because they directly influence relevant performance parameters like speed and turning angles in the different age categories. Additionally, steepness influences the tactical strategy of young and older athletes in GS, which is important within the context of skills development. The tactics used within a turn were different between young and older athletes which gives coaches new areas to work on with their athletes. Moreover, the finding that the older athletes spent more time in the gliding phase could be crucial for understanding technique and performance development in young athletes.

## Data Availability Statement

The datasets generated for this study are available on request to the corresponding author.

## Ethics Statement

The studies involving human participants were reviewed and approved by swissethics. Written informed consent from the participants' legal guardian/next of kin was not required to participate in this study in accordance with the national legislation and the institutional requirements.

## Author Contributions

SL and SG advised in planning the experimental setup and selection of the methods and materials. BB and MG did the YOUNG experiment. BB and RJ carried out the ELITE experiment. BB and RJ wrote the manuscript with the help of MR. FO did the main part of the data analysis. MR and SL supervised the project. BB and RJ contributed equally to this manuscript. All authors contributed to the article and approved the submitted version.

## Conflict of Interest

The authors declare that the research was conducted in the absence of any commercial or financial relationships that could be construed as a potential conflict of interest.
